# Traffic Offloading in Multicast Device-to-Device Cellular Networks: A Combinatorial Auction-Based Matching Algorithm

**DOI:** 10.3390/s20041128

**Published:** 2020-02-19

**Authors:** Devarani Devi Ningombam, Seokjoo Shin

**Affiliations:** 1 Department of Information Strategy, Electronics and Telecommunications Research Institute, 218 Gajeong-ro, Yuseong-gu, Daejeon 34129, Korea; devaraninin@gmail.com; 2 Department of Computer Engineering, Chosun University, 309 Pilmun-daero, Dong-gu, Gwangju 61452, Korea

**Keywords:** multicast device-to-device communication, cellular networks, fractional frequency reuse, Lagrange relaxation technique, combinatorial auction-based matching algorithm

## Abstract

In the last few years, multicast device-to-device (D2D) cellular networks has become a highly attractive area of research. However, a particularly challenging class of issues in this area is data traffic, which increases due to increase in video and audio streaming applications. Therefore, there is need for smart spectrum management policies. In this paper, we consider a fractional frequency reuse (FFR) technique which divides the whole spectrum into multiple sections and allows reusing of spectrum resources between the conventional cellular users and multicast D2D users in a non-orthogonal scenario. Since conventional cellular users and multicast D2D users shared same resources simultaneously, they generate severe data traffic and high communication overhead. To overcome these issues, in this paper we propose Lagrange relaxation technique to solve the non-convex problem and combinatorial auction-based matching algorithm to select the most desirable resource reuse partners by fulfilling the quality of service (QoS) requirements for both the conventional cellular users and multicast D2D users. Then, we formulate an optimization problem to maximize the overall system performance with least computational complexity. We demonstrate that our method can exploit a higher data rate, spectrum efficiency, traffic offload rate, coverage probability, and lower computational complexity.

## 1. Introduction

Device-to-device (D2D) communication is believed to be a promising solution to address the problem of data traffic and spectrum scarcity by enabling direct communication between proximity-based devices without passing traffic through the evolved node B (eNB) [1]. In an underlaying D2D communication scheme, potential D2D users reused available uplink cellular resources in a non-orthogonal manner. Thus, it provides higher data rates, coverage capacity, traffic offloading, spectral efficiency (SE), and energy efficiency (EE). However, the unprecedented growth of high data rate services, such as real-time video and multimedia sharing results in data snarl-ups. To deal with these issues, multicast D2D uplink cellular networks is introduced. Multicast D2D uplink cellular networks is a one-to-many technique which provides a means to send a single source data to multiple receivers on a network. From a technical point of view, multicast D2D can provide several advantages over unicast and broadcast D2D cellular networks in the following ways: enhance efficiency through traffic control, enable distributed applications, and optimize network performance through minimization of traffic redundancy [2]. However, multicast D2D communication generates problems of interference and high computational complexity. The main challenges are: interference from the cellular users to D2D receivers in a multicast D2D group, interference from the multicast D2D transmitter to the eNB and mutual interference between multicast D2D groups. To better handle these issues, reliable and efficient methods should be considered [3]. One of fundamental solutions is the content sharing-based multicast D2D communication [4], which can solve the resource allocation (RA) problem by considering physical attributes and social domains. These physical attributes of both cellular users and D2D users imply concerns to generate effective multicasting links. However, above the physical attributes, another main objective in multicast D2D communication is how to attain an optimal solution with proper channel assignment. To attain an optimal solution in multicast D2D underlaying cellular networks, resource-sharing optimization technique is one of the most efficient candidates. Another open challenge of multicast D2D cellular networks is the high data traffic. To address this issue, a variety of auction algorithms such as combinatorial auction, adaptive auction, and truthful auction algorithms have been adopted by 3rd Generation Partnership Project (3GPP) standardization [5,6,7]. In a combinatorial auction algorithm, bidders/participants bid on combinations of items [5]. This results in a scalable and manageable solution to spectrum resource allocation. In an adaptive auction algorithm, the seller adjusts parameters in response to the auction outcomes to increase the revenue [6], whereas in a truthful auction algorithm, buyers should bid according to their true price for the spectrum resources [7]. Furthermore, in D2D communications underlaying cellular networks, to achieve a tractable RA, several matching algorithms have been suggested. Maximum weighted bipartite matching algorithm solves the spectrum resource assignment problem and obtains the maximum matching degrees while guaranteeing least data traffic [8,9]. Here, the term matching degree is expressed using quality of service (QoS) requirements and network parameters. These works above analyze the resource reuse gain for both cellular and D2D users based on the users’ preferences. An energy-efficient matching algorithm in D2D cellular networks employs one-to-one matching problem under two-sided preferences [10]. The authors analyze the Gale-Shapley algorithm to solve the energy-efficient matching problem.

Most existing work on multicast D2D communications focuses on individual-based auction mechanisms to allocate available resources, without considering a fractional frequency reuse (FFR) scheme. Motivated by works in the literature, in this paper an FFR-based RA technique is proposed for multicast D2D communication underlaying uplink cellular networks. Based on the above-discussed issues, the main contributions of this paper are listed as follows:

- We introduce the FFR technique, in which the whole cell region is divided into two non-overlapping regions, namely the inner region (the region nearby the eNB) having low transmission power and the outer/edge region (the region far away from the eNB) having high transmission power. Furthermore, both regions are sectorized into three equivalent sections using three 120° directional antennas. In the FFR technique we have six different sections with different frequency sub-bands. The FFR technique can mitigate co-channel interference between cellular users and D2D users while reusing the same uplink cellular resources simultaneously, thus fulfills the demands of high data rate services for real-world applications.
- Then, to solve the non-convex issue in optimization problem, we introduce the Lagrangian relaxation technique (LRT). The LRT uses a Lagrangian multiplier (λ) to provide upper bounds of transmitting power.
- Moreover, to achieve scalable and manageable RA, we propose a combinatorial auction-based matching algorithm. A combinatorial auction-based matching algorithm can find the most promising resource reuse partner with minimal co-channel interference between cellular users and D2D users. For analysis, the conventional cellular users are considered as sellers, the D2D users requesting to access the cellular resources as buyers, and the eNB as auctioneer. We divide the proposed algorithm into two stages. In the first stage, the auctioneer identifies the set of prices broadcasted by cellular users and bidding price from the D2D users. Then, the auctioneer announces prices for resources to buyers and the buyers specify the resources they wish to reuse at the current price. When the buyer’s bidding price is equal to or more than the seller’s prices, the auctioneer will choose the buyer as the winner of the auction. The process will continue for all set of the bids that maximize the total system gain and auction will end when the auctioneer fails to find the bids that maximize the system gain. In the second stage, to find a stable matching with low data traffic and high reuse satisfaction rate, the matching algorithm is introduced. In the matching algorithm, we perform one-to-one matching of a resource to a multicast D2D group. A mapping function is defined to verify the matching algorithm.
- We have verified the benefits of our proposed algorithm by comparing the performances with different algorithms. The results show that our proposed algorithm offloads data traffic and maximizes the system performance with very less computational complexity.

We organize the remainder of this paper as follows. Section 2 presents related works. In Section 3, we present a system model. Problem formulation is presented in Section 4. Section 5 presents the performance analysis and discussion. A brief conclusion is presented in Section 6.

## 2. Related Work

Recently, multicast D2D communication in cellular networks has become an interesting topic due to its benefits of high data rate [11,12]. A single-rate multicasting scheme was presented in which a D2D transmitter sent the same information to multiple receivers in a multicast group. In this work, the authors formulated an optimization problem using a metaheuristic-tabu search algorithm to improve system throughput. A dynamic power control scheme for multicast D2D communications was analyzed in [13]. A game model with imperfect channel information was proposed by using a step-by-step interactive feedback mechanism. The results showed that the proposed scheme improved system throughput, bandwidth utilization, and SE. The limitation of the proposed scheme was that the dynamic behavior of device clustering was not considered. In [14], the authors studied the enhancements and challenges of multicasting by using a dynamic D2D user selection method. First, a tractable multicasting process was introduced to analyze the system in terms of coverage probability, the number of users covered, and system throughput. Finally, the authors formulated an optimization problem to achieve higher system capacity. The disadvantage of the study is that no generalized network-assisted D2D communication was considered, and thus low spectrum efficiency was achieved. A two-stage RA scheme of an OFDM network is proposed in [15] to improve the system spectral efficiency. In this paper, the authors considered a time window allocation stage and a subchannel reallocation stage. In [16], the authors proposed a coordinated multi-point transmission technique to obtain high SE. First, an auction algorithm with a joint beamforming method was analyzed. Finally, a power control scheme was discussed for efficient spectrum allocation in a coordinated multi-point transmission system, and an optimization problem was formulated to optimize the system utility. The disadvantage of the proposed method is that the algorithm repeated the updates of the beamforming vector, thus repetition phenomenon occurred. Fractional frequency reuse (FFR)-based spectrum auction algorithm was proposed in [17] to mitigate intra-cell interference and improve transmission efficiency for users far away from the eNB. Group reuse spectrum auction mechanism was proposed to obtain higher spectrum utilization efficiency. The disadvantage of the proposed scheme is that the computational complexity of the system increased with the increase in the number of users. Therefore, this method is not efficient for dense networks. In [18], the authors proposed a distributed antenna-based auction algorithm for cognitive radio networks to improve the spectrum efficiency. However, the proposed scheme could not provide an optimal solution for dense networks.

Data traffic offload is a challenge in D2D cellular networks since a large number of devices attempt to access a network simultaneously [19,20]. The works in [21,22,23,24,25,26] discussed various mobile data offloading techniques to improve system spectral efficiency. In [21], the authors proposed an efficient spectrum offloading technique in HetNets based on a reverse auction algorithm to maximize the benefit of a cellular service provider. A greedy offloading method was introduced to obtain tradeoff and spectrum efficiency with reduced computational complexity. However, the proposed scheme could not achieve an optimal solution when the size of the network is large. In [22], the authors proposed a base station switching-off mechanism for heterogeneous networks using an auction algorithm. In their work, a combinatorial auction framework was introduced by defining different objective functions, such as sellers and buyers bidding preferences and resource reuse payment criteria. The results showed that their proposed scheme attained efficient RA with energy savings. However, the tradeoff of traffic offloading and spectral efficiency was not discussed in the paper. A QoS-based incentive method was proposed in [23] to offload mobile data traffic. In this work, the authors considered diverse data patterns and the characteristics of various services to obtain a high QoS level for multiple mobile services. The results showed that their proposed scheme not only afforded higher social welfare but also provided truthfulness. However, the dynamic behavior of user demand and delay time was not analyzed. In [24], the authors proposed a randomized auction mechanism for D2D communication to offload data traffic. A trading network was developed by considering a group offloading method, and a randomized auction algorithm was further analyzed. The results showed that by guaranteeing spectral efficiency and truthfulness, their proposed scheme achieved higher system performance compared with existing methods. In [25], the authors proposed a spectrum auction algorithm for cellular network offloading. The dynamic characteristics of cellular network traffic demands were analyzed to minimize the costs sustained by the mobile network. Then an adaptive LRA was considered to solve the optimal reverse auction problem. The results showed that their proposed scheme met economical and networking criteria. However, the intra-cell interference was high, as no FFR scheme was considered. In [26], a multi-seller combinatorial auction algorithm was proposed to improve spectrum efficiency. At first, the seller introduced a reserve price and then used a greedy algorithm to determine the auction winner. Simulation results showed that the proposed scheme achieved high social welfare and spectrum efficiency. However, neither a grouped-based price determination technique nor the truthfulness of the seller was analyzed. Thus, the probability of fairness of resource assignment is low.

Finally, there has also been some work on matching based RA for D2D communication. The authors in [27] proposed social network-based content delivery with D2D communication using a matching technique. First, a Bayesian nonparametric model was introduced to analyze the probability of selecting users that had similar content interests. Then, an iterative matching algorithm was proposed to enhance the sum data rate of D2D users by guaranteeing the QoS requirements of the system. The limitation of this scheme is that an omnidirectional antenna was considered in the system model. This results in high intra-cell interference between cellular users and D2D users. Therefore, it would be better if the authors consider multiple directional antennas for both cellular and D2D networks. In [28], the authors proposed two effective clustering mechanisms, namely a Chinese restaurant process that assumed that data could be exchanged with other nearby data, and a distance-dependent Chinese restaurant process that basically analyzed distance and sequential data to model random partitions of nonconvertible data. However, in this work, the available frequency band was assigned to the users at the same time. This increases the interference between cellular users and D2D users, which degrades system performance. An optimal RA algorithm was introduced in [29] to maximize system performance rate. A weighted bipartite matching method was discussed, which avoids nonviable D2D users and allows multiple sharing of resources. The main drawback of the study is high co-channel interference.

## 3. System Model

**Figure 1:**
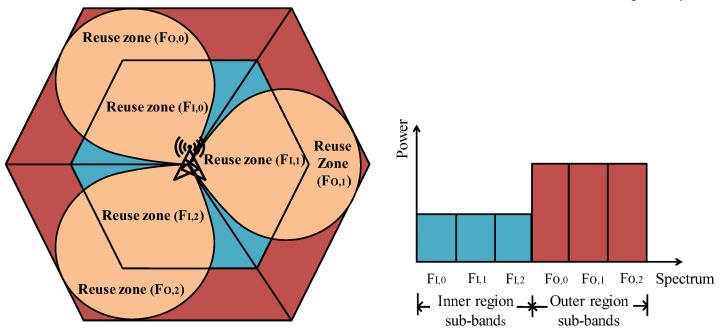
Spectrum partitioned in fractional frequency reuse (FFR) scheme using three 120°-directional antennas.

Considering the uplink of a cellular network, we introduced a densely deployed multicell cellular network controlled by an eNB located at the center of each cell. In this study, we consider fractional frequency reuse method as shown in Figure 1. The hexagonal cell area is divided into two regions namely inner and outer cell regions and both the regions are sectored into three sub-regions using three 120°-directional antennas. The spectrum sub-bands corresponding to inner and outer cell-regions are $\;F_{I,0},\;F_{I,1}$, $F_{I,2}$ and $F_{0,0},\;F_{0,1},\;F_{0,2}$, respectively as shown in Figure 1. As shown in Figure 1, partitioned FFR technique is considered, where the available spectrum band is first divided into two non-overlapping zones. The first part is dedicated to users located near the eNB, and the second part is for users located far away from the eNB (edge-cell users). Furthermore, these two zones are divided into six subzones. 

**Figure 2:**
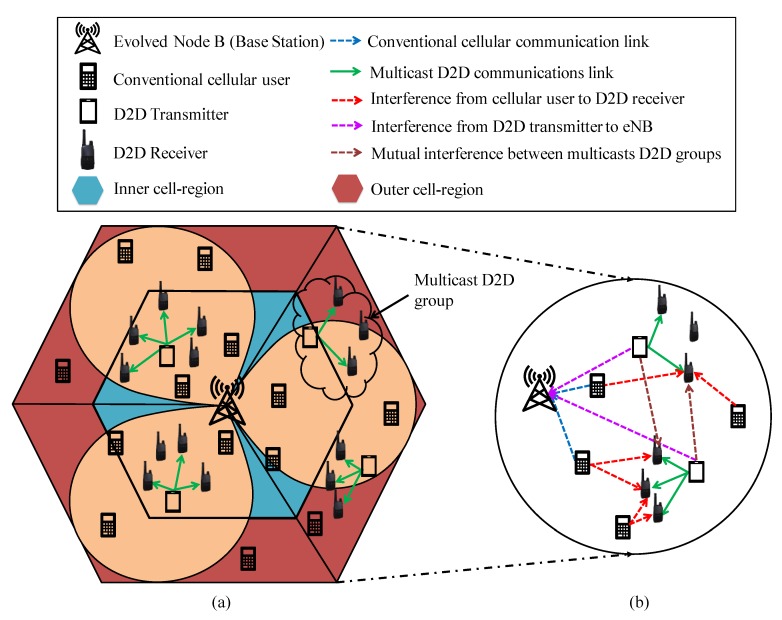
(**a**) System model for multicast device-to-device (D2D) communications, and (**b**) uplink interference scenarios.

We present the system model that adopted in the design of multicast D2D cellular networks and uplink interference scenarios in Figure 2a,b, respectively. For analysis, we consider that the conventional cellular users generally have a higher priority than multicast D2D users in each cell. We assume that the available uplink cellular resources can be reuse by multicast D2D users. In this paper, we consider a network in which there is a set of resources I={1, 2, …, I} owned by the eNB, a set of cellular users C={1, 2, …, C}, a set of D2D transmitters T={1, 2, …, T} and a set of D2D receivers ℛ={1, 2, …, R} that form a set of multicast D2D groups ℳ={1, 2, …, M}.

We assume that the potential D2D users are distributed in PPP form and consider the path-loss effect and log-normal shadowing propagation scenarios between the probable D2D transmitter and receivers in a multicast group. Therefore, the probability that the available receivers detect the signal transmitted by the D2D transmitter at a distance *l* is expressed as [10]

(1) $$F=P\left\{\frac{P_{T}\prod_{n}\emptyset_{n}}{l^{2\alpha }}\geq P_{Th}\right\},$$

where $P_{T}$ is the transmit power, $\emptyset_{n}$ is the independent random variable, α is the path loss exponent, and $P_{Th}$ is the threshold probability level. Using a Gaussian *Q*-function, (1) can be rewritten as [10]

(2) $$P_{R}=Q\left(\frac{1}{\vartheta }ln\left(\frac{P_{Th}\cdot l^{2\alpha }}{F}\right)\right),\;$$

where ϑ is the shadowing coefficient, and $P_{R}$ is the receiver power.

In a multicast D2D group, a multicast D2D transmitter can transmit the same data to multiple D2D receivers after analyzing the receiver’s channel demand criteria. Since the multicast D2D receivers have different channel qualities, the D2D transmitter will make a wise decision in favor of those receivers which can fulfill the channel criteria set by D2D transmitter. In this paper, we consider that the multicast D2D group *m* shares the same resource *i* with cellular user *c* and investigate the tradeoff between the signal-to-interference-plus-noise ratio (SINR) and RA to maximize the data rate while guaranteeing the QoS requirements. We define a binary variable $\delta_{c,m}^{i}$ to represent RA phenomenon with iϵI, cϵC and mϵℳ, such that

(3) $$\delta_{c,m}^{i}=\{\begin{array}{c} 1,\;\mathrm{resource}\;i\;\mathrm{is}\;\mathrm{allocated}\;\mathrm{to}\;\mathrm{multicast}\;D2D\;\mathrm{group}\;m\\ 0,\;\mathrm{otherwise} \end{array}$$

In order to reduce the power consumption of multicast D2D link, it is not necessary to transmit data at minimum rate. However, we consider a minimum SINR constraint to guarantee the QoS requirements for both cellular users and multicast D2D users. Therefore, the received SINR of cellular user *c* is derived as

(4) $$\gamma_{c}^{i}=\;\frac{P_{t}^{c}\cdot H_{c,eNB}}{\sigma^{2}+\;\sum_{m\epsilon ℳ}\delta_{c,m}^{i}\cdot \;P_{t}^{m}\cdot H_{m,eNB}}$$

Similarly, the received SINR of multicast D2D group *m* is listed as

(5) $$\gamma_{m}^{i}=\;\frac{P_{t}^{m}\cdot H_{m,c}}{\sigma^{2}+\;\delta_{c,m}^{i}\cdot \;P_{t}^{c}\cdot H_{m,c}+\sum_{m^{\prime }\neq m,m^{\prime }\epsilon ℳ}\delta_{c,m}^{i}\cdot \;P_{t}^{m^{\prime }}\cdot H_{m^{\prime },m}}$$

where $\gamma_{c}^{i}$ and $\gamma_{m}^{i}$ are the SINR received by the cellular user *c* and multicast D2D group *m* while using resource *i,* respectively, and $P_{t}^{c}$ and $P_{t}^{m}$ are the transmission power of cellular user *c* and multicast D2D group *m,* respectively. $H_{c,eNB}$ and $H_{m,c}$ are the channel gain between cellular user *c* and eNB, and between multicast D2D group *m* and cellular user *c*, respectively. $\sigma^{2}$ is the noise spectral density. Let $\gamma_{c}^{i}$ and $\gamma_{m}^{i}$ be the minimum SINR required for each cellular user and multicast D2D group that is set based on specific applications. For the interference term, the first combination $\sum_{m\epsilon ℳ}\delta_{c,m}^{i}\cdot \;P_{t}^{m}\cdot H_{m,eNB}$ in (4) is illustrated as the interference coming from co-channel D2D users. The second term $\delta_{m}^{i}\cdot \;P_{t}^{c}\cdot H_{m,c}$ in (5) is illustrated as the interference coming from co-channel cellular user, and the third combination $\sum_{m^{\prime }\neq m,m^{\prime }\epsilon ℳ}\delta_{c,m}^{i}\cdot \;P_{t}^{m^{\prime }}\cdot H_{m^{\prime },m}$ in (5) is illustrated as the interference coming from co-channel D2D users.

## 4. Problem Formulation

### 4.1. Proposed RA Algorithm without Lagrange Relaxation Technique (RA Wo LR)

In this work, we analyze the coverage probability of multicast D2D cellular networks. Here, the coverage probability is defined as the probability of a successful transmission of signal with a threshold SINR value. Therefore, the coverage probabilities of cellular user *c* ($\beta_{c}^{i}$) and multicast D2D group *m* ($\beta_{m}^{i}$) are derived as follows [6]:

(6) $$\beta_{c}^{i}=1-\left(1-\frac{1}{1+\frac{\left(\sum_{m\epsilon ℳ}\delta_{c,m}^{i}\cdot \;P_{t}^{m}\cdot H_{m,eNB}\right)\gamma_{T}}{P_{t}^{c}\cdot H_{c,eNB}}}\times e\left(-\frac{\sigma^{2}\cdot \gamma_{T}}{P_{t}^{c}\cdot H_{c,eNB}}\right)\right)$$

(7) $$\mathrm{and}\;\beta_{m}^{i}=1-\left(1-\frac{1}{1+\frac{\left(\delta_{c,m}^{i}\cdot \;P_{t}^{c}\cdot H_{m,c}+\sum_{m^{\prime }\neq m,m^{\prime }\epsilon ℳ}\delta_{c,m}^{i}\cdot \;P_{t}^{m^{\prime }}\cdot H_{m^{\prime },m}\right)\gamma_{T}}{P_{t}^{m}\cdot H_{m,c}}}\times e\left(-\frac{\sigma^{2}\cdot \gamma_{T}}{P_{t}^{m}\cdot H_{m,c}}\right)\right)$$

respectively, where $\gamma_{T}$ is the threshold SINR level. Then, the sum data rate can be expressed as

(8) $$R_{sum}^{i}=\left[R_{c}^{i}+\delta_{c,m}^{i}\cdot \;R_{m}^{i}\right]$$

We have

(9) $$R_{c}^{i}=\;log_{2}\left(1+\gamma_{c}^{i}\right)$$

(10) $$\mathrm{and}\;R_{m}^{i}=\;log_{2}\left(1+\gamma_{m}^{i}\right)$$

Substituting the value of $R_{c}^{i}$ and $R_{m}^{i}$ in (6), we have

(11) $$R_{sum}^{i}=log_{2}\left(1+\gamma_{c}^{i}\right)+\delta_{c,m}^{i}\cdot log_{2}\left(1+\gamma_{m}^{i}\right)$$

Using (4) and (5), (11) can be computed as

(12) $$R_{sum}^{i}=log_{2}\left(1+\frac{P_{t}^{c}\cdot H_{c,eNB}}{\sigma^{2}+\;\sum_{m\epsilon ℳ}\delta_{c,m}^{i}\cdot \;P_{t}^{m}\cdot H_{m,eNB}}\right)+\delta_{c,m}^{i}\cdot log_{2}\left(1+\;\frac{P_{t}^{m}\cdot H_{m,c}}{\sigma^{2}+\;\delta_{c,m}^{i}\cdot \;P_{t}^{c}\cdot H_{m,c}+\sum_{m^{\prime }\neq m,m^{\prime }\epsilon ℳ}\delta_{c,m}^{i}\cdot \;P_{t}^{m^{\prime }}\cdot H_{m^{\prime },m}}\right)$$

Therefore, the data rate maximization problem for $i^{th}$ resource can be expressed as

(13) $$S1.\;\underset{i\epsilon I}{\max}\sum_{c=1}^{C}\sum_{m=1}^{M}R_{sum}^{i}$$

such that

(14a) $$\delta_{c,m}^{i}=1,\;\;\forall \;i\;\epsilon \;I$$

(14b) $$\frac{P_{t}^{c}\cdot H_{c,eNB}}{\sigma^{2}+\;\sum_{m\epsilon ℳ}\delta_{c,m}^{i}\cdot \;P_{t}^{m}\cdot H_{m,eNB}}\;\geq \gamma_{c_{min}}^{i},\;\forall \;c\;\epsilon \;C$$

(14c) $$\frac{P_{t}^{m}\cdot H_{m,c}}{\sigma^{2}+\;\delta_{c,m}^{i}\cdot \;P_{t}^{c}\cdot H_{m,c}+\sum_{m^{\prime }\neq m,m^{\prime }\epsilon ℳ}\delta_{c,m}^{i}\cdot \;P_{t}^{m^{\prime }}\cdot H_{m^{\prime },m}}\geq \gamma_{m_{min}}^{i},\;\forall \;m\;\epsilon \;ℳ$$

(14d) $$P_{t_{min}}^{c}\;\leq \;P_{t}^{c}\leq P_{t_{max}}^{c},\;\forall \;c\;\epsilon \;C$$

(14e) $$P_{t_{min}}^{m}\;\leq P_{t}^{m}\leq P_{t_{max}}^{m},\;\forall \;m\;\epsilon \;ℳ$$

(14f) $$R_{c}^{i}\geq R_{Th},\;\;R_{m}^{i}\geq R_{Th}$$

where $\gamma_{c_{min}}^{i}$ and $\gamma_{m_{min}}^{i}$ are the minimum allowable SINR of cellular user *c* and multicast D2D group *m*, respectively, $P_{t_{max}}^{c}$ and $P_{t_{max}}^{m}$ are the maximum transmission power of cellular user *c* and multicast D2D group *m*, respectively, and $P_{t_{min}}^{c}$ and $P_{t_{min}}^{m}$ are the minimum transmission of the cellular user *c* and multicast D2D group *m*, respectively. Constraint (14a) indicates that $i^{th}$ resource is reused by $m^{th}$ multicast D2D group. Constraints (14b) and (14c) are the QoS requirements of cellular users and multicast D2D groups, respectively. Constraints (14d) and (14e) assure that the transmission power of cellular users and multicast D2D groups are within the lower and upper power levels, respectively. Hence, we can derive the performance gain for $i^{th}$ resource as

(15) $$W_{sum}^{i}=\max\left\{\sum_{c=1}^{C}\sum_{m=1}^{M}\left(R_{sum}^{i}-R_{c},\;0\right)\right\}$$

where $R_{c}$ is the data rate achieved by cellular user *c* without co-channel interference, i.e.,

(16) $$R_{c}=\;log_{2}\left(1+\frac{P_{t}^{c}\cdot H_{c,eNB}}{\sigma^{2}}\right)$$

Specifically, to optimize the sum data rate, it is necessary to check all the possible combinations between conventional cellular users and multicast D2D groups, as shown in (13). This phenomenon requires a long time period, and the computational complexity is O(C)+R×O(M). Moreover, the optimization problem formulated in (13) is an NP-hard problem. The pseudo code for the RA scheme is presented in Algorithm 1.

**Algorithm 1**: Pseudo Code for the RA Algorithm.
I = the set of resources
C = the set of cellular users
T = the set of D2D transmitters
ℛ = the set of D2D receivers
ℳ = the set of multicast D2D groups
Generate all randomly distributed cellular and D2D users’ locations within the cell of radius *y*
eNB transmits pilot signals to cellular and D2D users
**for** all cϵC, mϵℳ **do**
    Calculate distance between cellular users and D2D users and between D2D transmitter and nearly receivers
    Calculate the probability $P_{R}$ that the D2D receiver can detect the signal transmitted from the D2D transmitter
    **if** $\delta_{c,m}^{i}=1$, ∀ i ϵ I **then**
      Select the spectrum resource *i* which can share between cellular user and multicast D2D group
      Calculate $H_{c,eNB}$, $H_{m,eNB}$, $H_{m,c}$, and $H_{m^{\prime },m}$
      Calculate $\gamma_{c}^{i}$ and $\gamma_{m}^{i}$
      Calculate $\beta_{c}^{i}$ and $\beta_{m}^{i}$
      Calculate $R_{c}^{i}$ and $R_{m}^{i}$
      Formulate data rate maximization problem for $i^{th}$ resource as
        **if** $\gamma_{c}^{i}\;\geq \gamma_{c_{min}}^{i}$, $\gamma_{m}^{i}\;\;\geq \gamma_{m_{min}}^{i}$, $R_{c}^{i}\geq R_{Th}$, and $R_{m}^{i}\geq R_{Th}$ **then**  **S1.** $\underset{i\epsilon I}{\max}\sum_{c=1}^{C}\sum_{m=1}^{M}R_{sum}^{i}$, ∀ cϵC, mϵℳ  
Calculate $W_{sum}^{i}$
    **else**    $\delta_{c,m}^{i}=0$
    end **if**
    **else**
    Select the spectrum band *i’*,$\;i^{\prime }\;\epsilon \;I$    Calculate $H_{c,eNB}$, $H_{m,eNB}$, $H_{m,c}$, $H_{m^{\prime },m}$, $\gamma_{c}^{i^{\prime }}$, $\gamma_{m}^{i^{\prime }}$ $\beta_{c}^{i^{\prime }}$, $\beta_{m}^{i^{\prime }}$, $R_{c}^{i^{\prime }}$, and $R_{m}^{i^{\prime }}$    end **if**end **for**Check for all available resources that fulfill the reuse criteria


### 4.2. Proposed RA Algorithm with Lagrange Relaxation Technique (RA W LR)

In the data rate optimization problem formulated in (13), the computational complexity increases with the increase in the density of the network. This results in large overhead generation and difficult to find an optimal solution [30]. To solve this issue, we discuss a Lagrangian relaxation method which can find the upper bounds that are as close as possible to the optimal solution. In the Lagrangian relaxation method, we introduce Lagrange multipliers $\lambda =\left\{\lambda_{x},\;x=1,\;2,\;\ldots ,\;k\right\}$ to convert a set of constraints into the objective function. Specifically, for any λ≥0, the method attains an upper bound for the optimal solution. The power constraints in (14.d) and (14.e) are discussed in terms of Lagrangian multiplier λ as listed below [31]:

(17) $$f\left(P_{t}^{c},\;P_{t}^{m},\;\lambda_{1},\;\lambda_{2}\right)=R_{c}^{i}+R_{m}^{i}+\;\lambda_{1}\left(P_{t}^{c}-P_{t_{min}}^{c}+P_{t}^{m}-P_{t_{min}}^{m}\right)-\lambda_{2}\left(P_{t}^{c}-P_{t_{max}}^{c}+P_{t}^{m}-P_{t_{max}}^{m}\right)$$

Using the Karush–Kuhn–Tucker (KKT) method, we define the dual function of Equation (17) as follows:

(18) $$\min\left\{f\left(P_{t}^{c},\;P_{t}^{m},\;\lambda_{1},\;\lambda_{2}\right)\right\}$$

Therefore, the first-order optimality conditions are obtained as

(19) $$\frac{\partial f\left(P_{t}^{c},\;P_{t}^{m},\;\lambda_{1},\;\lambda_{2}\right)}{\partial P_{t}^{c}}=0$$

(20) $$\frac{\partial f\left(P_{t}^{c},\;P_{t}^{m},\;\lambda_{1},\;\lambda_{2}\right)}{\partial P_{t}^{c}}=\frac{\partial f\left(R_{c}^{i}+R_{m}^{i}+\;\lambda_{1}\left(P_{t}^{c}-P_{t_{min}}^{c}+P_{t}^{m}-P_{t_{min}}^{m}\right)\right)}{\partial P_{t}^{c}}-\frac{\partial f\left(\lambda_{2}\left(P_{t}^{c}+P_{t_{max}}^{c}-P_{t}^{m}+P_{t_{max}}^{m}\right)\right)}{\partial P_{t}^{c}}=0$$

(21) $$\frac{\partial f\left(P_{t}^{c},\;P_{t}^{m},\;\lambda_{1},\;\lambda_{2}\right)}{\partial P_{t}^{c}}=\frac{\partial f\left(R_{c}^{i}\right)}{\partial P_{t}^{c}}+\frac{\partial f\left(R_{m}\right)}{\partial P_{t}^{c}}+\frac{\partial f\left(\;\lambda_{1}\left(P_{t}^{c}-P_{t_{min}}^{c}+P_{t}^{m}-P_{t_{min}}^{m}\right)\right)}{\partial P_{t}^{c}}-\frac{\partial f\left(\;\lambda_{2}\left(P_{t}^{c}-P_{t_{max}}^{c}+P_{t}^{m}-P_{t_{max}}^{m}\right)\right)}{\partial P_{t}^{c}}=0$$

We have,

(22) $$\frac{\partial f\left(R_{c}^{i}\right)}{\partial P_{t}^{c}}=\frac{\sigma^{2}+\;\sum_{m\epsilon ℳ}\delta_{c,m}^{i}\cdot \;P_{t}^{m}\cdot H_{m,eNB}}{P_{t}^{c}\cdot H_{c,eNB}}\cdot \frac{H_{c,eNB}}{\sigma^{2}+\;\sum_{m\epsilon ℳ}\delta_{c,m}^{i}\cdot \;P_{t}^{m}\cdot H_{m,eNB}}=\frac{1}{P_{t}^{c}}$$

(23) $$\frac{\partial f\left(R_{m}^{i}\right)}{\partial P_{t}^{c}}=\delta_{m}^{i}\left[\frac{\sigma^{2}+\;\delta_{c,m}^{i}\cdot \;P_{t}^{c}\cdot H_{m,c}+\sum_{m^{\prime }\neq m,m^{\prime }\epsilon ℳ}\delta_{c,m}^{i}\cdot \;P_{t}^{m^{\prime }}\cdot H_{m^{\prime },m}}{P_{t}^{m}\cdot H_{m,c}}\times \frac{-P_{t}^{m}\cdot H_{m,c}\cdot \left(\delta_{c,m}^{i}\cdot H_{m,c}\right)}{{\left(\sigma^{2}+\;\delta_{c,m}^{i}\cdot \;P_{t}^{c}\cdot H_{m,c}+\sum_{m^{\prime }\neq m,m^{\prime }\epsilon ℳ}\delta_{c,m}^{i}\cdot \;P_{t}^{m^{\prime }}\cdot H_{m^{\prime },m}\right)}^{2}}\right]$$

(24) $$\frac{\partial f\left(R_{m}^{i}\right)}{\partial P_{t}^{c}}=\frac{-{\left(\delta_{c,m}^{i}\right)}^{2}\cdot H_{m,c}}{\sigma^{2}+\;\delta_{c,m}^{i}\cdot \;P_{t}^{c}\cdot H_{m,c}+\sum_{m^{\prime }\neq m,m^{\prime }\epsilon ℳ}\delta_{c,m}^{i}\cdot \;P_{t}^{m^{\prime }}\cdot H_{m^{\prime },m}}$$

(25) $$\frac{\partial f\left(\;\lambda_{1}\left(P_{t}^{c}-P_{t_{min}}^{c}+P_{t}^{m}-P_{t_{min}}^{m}\right)\right)}{\partial P_{t}^{c}}=\lambda_{1}$$

(26) $$\mathrm{and}\;\frac{\partial f\left(\;\lambda_{2}\left(P_{t}^{c}-P_{t_{max}}^{c}+P_{t}^{m}-P_{t_{max}}^{m}\right)\right)}{\partial P_{t}^{c}}=\lambda_{2}$$

Substituting the values of $\frac{\partial f\left(R_{c}^{i}\right)}{\partial P_{t}^{c}}$, $\frac{\partial f\left(R_{m}^{i}\right)}{\partial P_{t}^{c}}$, $\frac{\partial f\left(\;\lambda_{1}\left(P_{t}^{c}-P_{t_{min}}^{c}+P_{t}^{m}-P_{t_{min}}^{m}\right)\right)}{\partial P_{t}^{c}}$, and $\frac{\partial f\left(\;\lambda_{2}\left(P_{t}^{c}-P_{t_{max}}^{c}+P_{t}^{m}-P_{t_{max}}^{m}\right)\right)}{\partial P_{t}^{c}}$ in (21), we have

(27) $$\frac{\partial f\left(P_{t}^{c},\;P_{t}^{m},\;\lambda_{1},\;\lambda_{2}\right)}{\partial P_{t}^{c}}=\frac{1}{P_{t}^{c}}-\frac{{\left(\delta_{c,m}^{i}\right)}^{2}\cdot H_{m,c}}{\sigma^{2}+\;\delta_{c,m}^{i}\cdot \;P_{t}^{c}\cdot H_{m,c}+\sum_{m^{\prime }\neq m,m^{\prime }\epsilon ℳ}\delta_{c,m}^{i}\cdot \;P_{t}^{m^{\prime }}\cdot H_{m^{\prime },m}}+\lambda_{1}-\lambda_{2}=0$$

(28) $$\lambda_{1}-\lambda_{2}=\frac{{\left(\delta_{c,m}^{i}\right)}^{2}\cdot H_{m,c}}{\sigma^{2}+\;\delta_{c,m}^{i}\cdot \;P_{t}^{c}\cdot H_{m,c}+\sum_{m^{\prime }\neq m,m^{\prime }\epsilon ℳ}\delta_{c,m}^{i}\cdot \;P_{t}^{m^{\prime }}\cdot H_{m^{\prime },m}}-\frac{1}{P_{t}^{c}}$$

(29) $$\lambda_{1}-\lambda_{2}=\frac{{\left(\delta_{c,m}^{i}\right)}^{2}\cdot H_{m,c}\cdot P_{t}^{c}-\left(\sigma^{2}+\;\delta_{c,m}^{i}\cdot \;P_{t}^{c}\cdot H_{m,c}+\sum_{m^{\prime }\neq m,m^{\prime }\epsilon ℳ}\delta_{c,m}^{i}\cdot \;P_{t}^{m^{\prime }}\cdot H_{m^{\prime },m}\right)}{P_{t}^{c}\times \left(\sigma^{2}+\;\delta_{c,m}^{i}\cdot \;P_{t}^{c}\cdot H_{m,c}+\sum_{m^{\prime }\neq m,m^{\prime }\epsilon ℳ}\delta_{c,m}^{i}\cdot \;P_{t}^{m^{\prime }}\cdot H_{m^{\prime },m}\right)}$$

Additionally,

(30) $$\frac{\partial f\left(P_{t}^{c},\;P_{t}^{m},\;\lambda_{1},\;\lambda_{2}\right)}{\partial P_{t}^{m}}=0$$

(31) $$\frac{\partial f\left(P_{t}^{c},\;P_{t}^{m},\;\lambda_{1},\;\lambda_{2}\right)}{\partial P_{t}^{m}}=\frac{\partial f\left(R_{c}+R_{m}+\;\lambda_{1}\left(P_{t}^{c}-P_{t_{min}}^{c}+P_{t}^{m}-P_{t_{min}}^{m}\right)-\lambda_{2}\left(P_{t}^{c}-P_{t_{max}}^{c}+P_{t}^{m}-P_{t_{max}}^{m}\right)\right)}{\partial P_{t}^{m}}=0$$

(32) $$\frac{\partial f\left(P_{t}^{c},\;P_{t}^{m},\;\lambda_{1},\;\lambda_{2}\right)}{\partial P_{t}^{m}}=\frac{\partial f\left(R_{c}\right)}{\partial P_{t}^{m}}+\frac{\partial f\left(R_{m}\right)}{\partial P_{t}^{m}}+\frac{\partial f\left(\;\lambda_{1}\left(P_{t}^{c}-P_{t_{min}}^{c}+P_{t}^{m}-P_{t_{min}}^{m}\right)\right)}{\partial P_{t}^{m}}-\frac{\partial f\left(\;\lambda_{2}\left(P_{t}^{c}-P_{t_{max}}^{c}+P_{t}^{m}-P_{t_{max}}^{m}\right)\right)}{\partial P_{t}^{m}}=0$$

We have,

(33) $$\frac{\partial f\left(R_{c}^{i}\right)}{\partial P_{t}^{m}}=\frac{\sigma^{2}+\;\sum_{m\epsilon ℳ}\delta_{c,m}^{i}\cdot \;P_{t}^{m}\cdot H_{m,eNB}}{P_{t}^{c}\cdot H_{c,eNB}}\times \frac{-P_{t}^{c}\cdot H_{c,eNB}\left(\sum_{m\epsilon ℳ}\delta_{c,m}^{i}\cdot \;H_{m,eNB}\right)}{{\left(\sigma^{2}+\;\sum_{m\epsilon ℳ}\delta_{c,m}^{i}\cdot \;P_{t}^{m}\cdot H_{m,eNB}\right)}^{2}}=\frac{-\sum_{m\epsilon ℳ}\delta_{c,m}^{i}\cdot \;H_{m,eNB}}{\sigma^{2}+\;\sum_{m\epsilon ℳ}\delta_{c,m}^{i}\cdot \;P_{t}^{m}\cdot H_{m,eNB}}$$

(34) $$\frac{\partial f\left(R_{m}^{i}\right)}{\partial P_{t}^{m}}=\delta_{m}^{i}\left[\frac{\sigma^{2}+\;\delta_{c,m}^{i}\cdot \;P_{t}^{c}\cdot H_{m,c}+\sum_{m^{\prime }\neq m,m^{\prime }\epsilon ℳ}\delta_{c,m}^{i}\cdot \;P_{t}^{m^{\prime }}\cdot H_{m^{\prime },m}}{P_{t}^{m}\cdot H_{m,c}}\right]\times \frac{\left(\sigma^{2}+\;\delta_{c,m}^{i}\cdot \;P_{t}^{c}\cdot H_{m,c}+\sum_{m^{\prime }\neq m,m^{\prime }\epsilon ℳ}\delta_{c,m}^{i}\cdot \;P_{t}^{m^{\prime }}\cdot H_{m^{\prime },m}\right)H_{m,c}}{{\left(\sigma^{2}+\;\delta_{c,m}^{i}\cdot \;P_{t}^{c}\cdot H_{m,c}+\sum_{m^{\prime }\neq m,m^{\prime }\epsilon ℳ}\delta_{c,m}^{i}\cdot \;P_{t}^{m^{\prime }}\cdot H_{m^{\prime },m}\right)}^{2}}$$

(35) $$\frac{\partial f\left(R_{m}\right)}{\partial P_{t}^{m}}=\frac{1}{P_{t}^{m}}$$

(36) $$\frac{\partial f\left(\;\lambda_{1}\left(P_{t}^{c}-P_{t_{min}}^{c}+P_{t}^{m}-P_{t_{min}}^{m}\right)\right)}{\partial P_{t}^{m}}=\lambda_{1}$$

(37) $$\mathrm{and}\;\frac{\partial f\left(\;\lambda_{2}\left(P_{t}^{c}-P_{t_{max}}^{c}+P_{t}^{m}-P_{t_{max}}^{m}\right)\right)}{\partial P_{t}^{m}}=\lambda_{2}$$

Substituting the values of $\frac{\partial f\left(R_{c}^{i}\right)}{\partial P_{t}^{m}}$, $\frac{\partial f\left(R_{m}^{i}\right)}{\partial P_{t}^{m}}$, $\frac{\partial f\left(\;\lambda_{1}\left(P_{t}^{c}-P_{t_{min}}^{c}+P_{t}^{m}-P_{t_{min}}^{m}\right)\right)}{\partial P_{t}^{m}}$, and $\frac{\partial f\left(\;\lambda_{2}\left(P_{t}^{c}-P_{t_{max}}^{c}+P_{t}^{m}-P_{t_{max}}^{m}\right)\right)}{\partial P_{t}^{m}}=\lambda_{2}$ in (30), we have

(38) $$\frac{\partial f\left(P_{t}^{c},\;P_{t}^{m},\;\lambda_{1},\;\lambda_{2}\right)}{\partial P_{t}^{m}}=\frac{-\sum_{m\epsilon ℳ}\delta_{c,m}^{i}\cdot \;H_{m,eNB}}{\sigma^{2}+\;\sum_{m\epsilon ℳ}\delta_{c,m}^{i}\cdot \;P_{t}^{m}\cdot H_{m,eNB}}+\frac{1}{P_{t}^{m}}+\lambda_{1}-\lambda_{2}=0$$

(39) $$\lambda_{1}-\lambda_{2}=\frac{\sum_{m\epsilon ℳ}\delta_{c,m}^{i}\cdot \;H_{m,eNB}}{\sigma^{2}+\;\sum_{m\epsilon ℳ}\delta_{c,m}^{i}\cdot \;P_{t}^{m}\cdot H_{m,eNB}}-\frac{1}{P_{t}^{m}}$$

(40) $$\lambda_{1}-\lambda_{2}=\frac{P_{t}^{m}-\sum_{m\epsilon ℳ}\delta_{c,m}^{i}\cdot \;H_{m,eNB}}{\left(\sigma^{2}+\;\sum_{m\epsilon ℳ}\delta_{c,m}^{i}\cdot \;P_{t}^{m}\cdot H_{m,eNB}\right)P_{t}^{m}}$$

In this case, four possible conditions exist, as given below

- Case 1: $\lambda_{1}=0$ and $\lambda_{2}=0$ means that both performances constrains of cellular user and D2D user are invalid.
- Case 2: $\lambda_{1}=0$ and $\lambda_{2}\neq 0$ means that the sub-optimal solution exits when $P_{t}^{c}=P_{t_{min}}^{c}$ and $P_{t}^{m}=P_{t_{min}}^{m}$.
- Case 3: $\lambda_{1}\neq 0$ and $\lambda_{2}=0$ means that the sub-optimal solution exits when $P_{t}^{c}=P_{t_{max}}^{c}$ and $P_{t}^{m}=P_{t_{max}}^{m}$.
- Case 4: $\lambda_{1}\neq 0$ and $\lambda_{2}\neq 0$ means that the sub-optimal solution exits when both $P_{t}^{c}=P_{t_{min}}^{c}$, $P_{t}^{m}=P_{t_{min}}^{m}$ and $P_{t}^{c}=P_{t_{max}}^{c}$ and $P_{t}^{m}=P_{t_{max}}^{m}$.

### 4.3. Proposed RA with Combinatorial Auction-based Matching Algorithm (RA W CA)

To support a comparably scalable and manageable RA, this paper considers a combinatorial auction-based matching algorithm. In our combinatorial action algorithm, we assume that the bidding price for spectrum resources set by the eNB is known to all users and bidding mechanism follows sealed-bid first price auction method. In the sealed-bid first price auction method, a bidder submits the bid to the auctioneer in such a way that other opponent bidders could not see the submitted bid. Therefore, estimation costs of different bidders are unknown to each other. If the bidder’s submitted bid is highest among all the submitted bids to the auctioneer, the highest bidder wins the bid and will charge the amount he bid. We also assume that the proposed combinatorial auction algorithm allows one-to-one bargaining scenario. Several considerations have made to implement the combinatorial auction algorithm and introduced some of the main notations below [26]. We assume that equal power is assigned to each allocated subchannel, and the channel gains of cellular users and multicast D2D users are different. We also consider that the auction mechanism depends on the utility of resources that favors for the channel gain from multicast D2D users.

We divide the combinatorial auction-based matching algorithm into two stages. In the first stage, combinatorial auction algorithm is performed by considering the set of prices broadcasted by the cellular users to enable reuse of their resources and D2D bidding prices. At this point, it is worth mentioning that the main task of the auctioneer is to update the associated bids that maximizes the system revenue.

Suppose that when auctioning an uplink resource *i* to multicast D2D group *m*, D2D receivers are analyzed with the same probability distribution. Then, the bidding price of the resource *i* set by the eNB is known to all D2D receivers in the multicast group *m.* However, each D2D receivers have different estimations and are unknown to each other. We analyze the main characteristics used in our experiment as follows:

- Resource reuse request *Q*: A D2D transmitter belonging to a multicast group send request Q= (1, 2, …, $Q_{r}$) to the auctioneer over wireless channel. The auctioneer acknowledges the bidding message for each of the requests that meet the requirements of eNB.
- Bidding price $S_{eNB}^{i}$: The price to be pay by the buyer for reusing the resource *i*. Considering the same bidding price of all available resources, $S_{eNB}^{i}$ can be calculated as
  (41) $$S_{eNB}^{i}=\;\sum_{i\epsilon I}s_{eNB}^{i}=\sum_{i\epsilon I}s_{m}^{i},\;\forall \;m\epsilon ℳ$$
- Resource reuse gain $G_{eNB}^{i}$: The resource reuse gain for $i^{th}$ resource can be calculated as
  (42) $$G_{eNB}^{i}=W_{sum}^{i}-S_{eNB}^{i}$$
  Therefore, the sum resource reuse gain can be denoted as
  (43) $$G_{sum}=\;\sum_{i=1}^{I}\sum_{m=1}^{M}\delta_{m}^{i}\cdot \;G_{eNB}^{i}$$
- Auctioneer’s gain $G_{a}$: The auctioneer’s gain can be calculated as
  (44) $$G_{a}=\sum_{i=1}^{I}\sum_{c=1}^{C}\delta_{m}^{i}\cdot \;S_{eNB}^{i}$$
  Hence, the performance gain is expressed as
  (45) $$G=\;G_{sum}+G_{a}$$
  (46) $$G=\sum_{i=1}^{I}\sum_{m=1}^{M}\delta_{m}^{i}\cdot \;G_{eNB}^{i}+\sum_{i=1}^{I}\sum_{c=1}^{C}\delta_{m}^{i}\cdot \;S_{eNB}^{i}$$
  The optimization problem for maximizing the performance gain can be expressed as
  (47) $$S2.\;\max\sum_{i=1}^{I}\sum_{c=1}^{C}\sum_{m=1}^{M}G$$
  such that
  (48a) $$\sum_{m=1}^{M}\delta_{c,m}^{i}\epsilon \;\left\{0,1\right\},\;\forall \;i\;\epsilon \;I$$
  (48b) $$\frac{P_{t}^{c}\cdot H_{c,eNB}}{\sigma^{2}+\;\sum_{m\epsilon M}\delta_{c,m}^{i}\cdot \;P_{t}^{m}\cdot H_{m,eNB}}\;\geq \gamma_{c_{min}}^{i},\;\forall \;c\;\epsilon \;C$$
  (48c) $$\frac{P_{t}^{m}\cdot H_{m,c}}{\sigma^{2}+\;\delta_{c,m}^{i}\cdot \;P_{t}^{c}\cdot H_{m,c}+\sum_{m^{\prime }\neq m,m^{\prime }\epsilon M}\delta_{c,m}^{i}\cdot \;P_{t}^{m^{\prime }}\cdot H_{m^{\prime },m}}\geq \gamma_{m_{min}}^{i},\;\forall \;m\;\epsilon \;ℳ$$
  (48d) $$P_{t_{min}}^{c}\;\leq \;P_{t}^{c}\leq P_{t_{max}}^{c},\;\forall \;c\;\epsilon \;C$$
  (48e) $$P_{t_{min}}^{m}\;\leq P_{t}^{m}\leq P_{t_{max}}^{m},\;\forall \;m\;\epsilon \;ℳ$$

The pseudo code for the auction algorithm is given in Algorithm 2.
**Algorithm 2**: Pseudo Code for the Auction Algorithm.Q = the set of all requests from multicast D2D groups to reuse cellular resources
Set $P_{t}^{c}=P_{t_{max}}^{c}$ and $P_{t_{min}}^{m}\;\leq P_{t}^{m}\leq P_{t_{max}}^{m}$
All cellular users broadcast a set of prices **for** all cϵC, mϵℳ **do**
Multicast D2D transmitters start auction algorithm simultaneously **if**$S_{eNB}$≥$s_{m}$, **then**Allocates the resources to the multicast groups Calculate $\gamma_{c}$, $\gamma_{m}$, $R_{c}$, and $R_{m}$
Calculate $G_{sum}$ and *G*
Formulate the performance gain optimization problem as **S2.** $\max\sum_{i=1}^{I}\sum_{c=1}^{C}\sum_{m=1}^{M}G$
**else**$S_{eNB}$<$s_{m}$end **if**
end **for**

**Figure 3:**
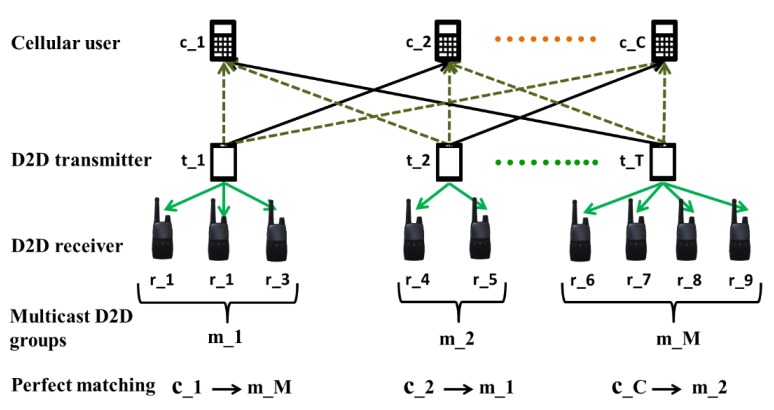
Selection of resource reuse matching between conventional cellular users and multicast D2D groups.

To achieve a tractable RA, in the second stage, a one-to-many matching scenario is performed to maximize the offloading traffic. The matching mechanism is also known as mapping, which maps from a cellular user’s (seller) resource to a multicast D2D group (buyer). To characterize the mapping mechanism, we defined a mapping function $\beta_{1}$ that satisfies the weighted mapping condition. According to the mutual preferences and weights between sellers and buyers, resource reusing partnerships are chosen. Hence in this matching scenario, we consider the sellers and buyers from stage one that maximized the system revenue under constraints of QoS and transmission power. The matching model is said to be successful when the mutual preferences between the sellers and buyers are achieved with the lowest interference levels. Selection of uplink resource reuse matching between the conventional cellular users and multicast D2D groups is shown in Figure 3. 

**Definition 1:** For the matching problem between the sellers and buyers, we defined a mapping function $\beta_{1}$. The mapping function $\beta_{1}$ should satisfy the following criteria to achieve optimal RA with the best system revenue gain,

- $\beta_{1}\left(c\right)\epsilon \;M\cup^{\;}\left\{C\right\},\;c\;\epsilon \;C$
- $\beta_{1}\left(m\right)\epsilon \;C\cup^{\;}\left\{M\right\},\;\forall \;m\;\epsilon \;ℳ$
- $\left|\beta_{1}\left(c\right)\right|\geq \;\beta_{1}\left(m\right),\;\forall \;c\;\epsilon \;C,\;m\;\epsilon \;ℳ$
- $\beta_{1}\left(c\right)=m$, iff $\beta_{1}\left(m\right)=c$

where $\beta_{1}\left(m\right)$ is the mapping function of the $m^{th}$ multicast D2D group and $\left|\beta_{1}\left(c\right)\right|\geq \;\beta_{1}\left(m\right)$ signifies that the number of cellular resources that can be reused by the multicast D2D users should be greater than or equal to the number of available multicast D2D groups. Then, m sends a request to *c* to reuse the resource *I* based on its preference.

By considering the above matching scenarios, we analyze the weighted performance gain as follows [32]

(49) $$G_{w}=C\times M\times \frac{G}{\sum_{i=1}^{I}\sum_{c=1}^{C}\sum_{m=1}^{M}G}$$

Therefore, the optimization problem in (47) can be reformulated as follows

(50) $$S3.\;\max\sum_{i=1}^{I}\sum_{c=1}^{C}\sum_{m=1}^{M}G_{w}$$

such that

(51a) $$\delta_{c,m}^{i}=1,\;\forall \;i\;\epsilon \;I$$

(51b) $$\frac{P_{t}^{c}\cdot H_{c,eNB}}{\sigma^{2}+\;\sum_{m\epsilon ℳ}\delta_{c,m}^{i}\cdot \;P_{t}^{m}\cdot H_{m,eNB}}\;\geq \gamma_{c_{min}}^{i},\;\forall \;c\;\epsilon \;C$$

(51c) $$\frac{P_{t}^{m}\cdot H_{m,c}}{\sigma^{2}+\;\delta_{c,m}^{i}\cdot \;P_{t}^{c}\cdot H_{m,c}+\sum_{m^{\prime }\neq m,m^{\prime }\epsilon ℳ}\delta_{c,m}^{i}\cdot \;P_{t}^{m^{\prime }}\cdot H_{m^{\prime },m}}\geq \gamma_{m_{min}}^{i},\;\forall \;m\;\epsilon \;ℳ$$

(51d) $$P_{t_{min}}^{c}\;\leq \;P_{t}^{c}\leq P_{t_{max}}^{c},\;\forall \;c\;\epsilon \;C$$

(51e) $$P_{t_{min}}^{m}\;\leq P_{t}^{m}\leq P_{t_{max}}^{m},\;\forall \;m\;\epsilon \;ℳ$$

In this scenario, it is beneficial to analyze the spectrum utilization efficiency of the system. The spectrum utilization efficiency can be defined as the ratio of the number of frequency bands occupied by the multicast D2D groups ($S_{D2D}$) to the total number of available frequency bands ($S_{total}$), i.e.,

(52) $$S_{eff}=\frac{S_{D2D}}{S_{total}}$$

The pseudo code of the proposed combinatorial auction-based matching algorithm for multicast D2D cellular networks is given in Algorithm 3.
**Algorithm 3**: Pseudo Code for the Auction-based Matching Algorithm.**while**$\beta_{1}\left(c\right)\neq 0$, $\beta_{1}\left(m\right)\neq 0$**do****for each**$\delta_{c,m}=1$**do****if**$S_{eNB}$≥$s_{m}$, **then**Calculate the weights of each multicast D2D groups **if**$\sum_{i=1}^{I}\sum_{c=1}^{C}\sum_{m=1}^{M}G>0$**then**Calculate $G_{w}$
Formulate the weighted performance gain optimization problem **S3.** $\max\sum_{i=1}^{I}\sum_{c=1}^{C}\sum_{m=1}^{M}G_{w}$
Calculate $S_{eff}$
**else**$\sum_{i=1}^{I}\sum_{c=1}^{C}\sum_{m=1}^{M}G\leq 0$end **if**
**else**$S_{eNB}$<$s_{m}$end **if**
$\delta_{c,m}=0$end **for**end **while**

### 4.4. Computational Complexity Analysis

In the proposed scheme, a near optimal performance gain is obtained with least data traffic and high spectrum efficiency. Specifically, while allocating resources to each promising cellular user, the eNB considers matching the preferences of both sides. Hence, the complexity of the process is O(C−M). Moreover, with the resource reuse mapping mechanism, cellular users choose the most effective multicast D2D groups that have similar preferences. The complexity of such a process is $R\times O\left(\frac{M}{N}\right)$. Therefore, the overall computational complexity of network is $O\left(C-M\right)+R\times O\left(\frac{M}{N}\right)$ instead of O(C)+R×O(M).

## 5. Performance Discussion

### 5.1. Simulation Environment

In our simulation, we consider a multicell cellular network, where conventional cellular users and D2D users are distributed randomly within each cell with radius *y* and eNB is situated at the center of each cell. We assume an uplink channel bandwidth 15 MHz [33], which is equally shared within *C* cellular users. This paper considers the path loss exponent α, which ranges from 3 to 5, the path loss constant with value 0.01, the log-normal distribution with 8 dB deviation, and the additive white Gaussian noise with power –174 dBm for all channels. The maximum transmission power for all cellular users and D2D users are 24 dBm and 20 dBm, respectively. In addition, to maintain the average coverage probability, we consider different densities of the concurrent cellular users and multicast D2D users as 0.0001 $m^{-2}$ and 0.0004 $m^{-2}$, respectively. In this study, we analyze the performance of multicast D2D communications, where the D2D receivers are randomly distributed in a defined region, and transmission is allowed within the range of 5–100 m. The proposed scheme was implemented in a Monte-Carlo simulator and the main simulation parameters and values have been illustrated in Table 1.

### 5.2. Simulation Results

In this subsection, the numerical results obtained from the expressions derived for combinatorial auction-based matching algorithm were analyzed to illustrate the validity of the proposed algorithm. We have compared our proposed algorithm with the RA algorithms presented in [5,9]. In the resource allocation schemes presented in [5,9], there is absence of FFR and spectrum partitioning techniques i.e., one omnidirectional antenna is used to cover the entire cell region.

**Figure 4:**
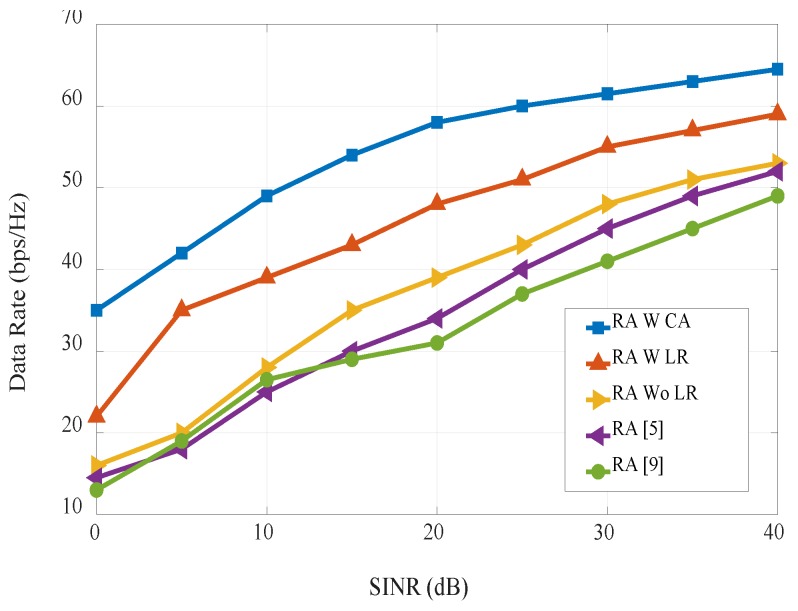
Data rate vs. signal-to-interference-plus-noise ratio (SINR) performance.

In Figure 4, the data rate with varying SINR is depicted and compared for different RA schemes. It can be observed that the proposed RA scheme with combinatorial suction-based matching algorithm resulted in a higher data rate than other schemes over the entire SINR range considered. This is because the proposed scheme maintained the upper and lower bounds transmission powers of each cellular and multicast D2D groups using the Lagrange relaxation method. Moreover, the co-channel interference was minimized with the use of three directional antennas and cell partitioning method. It can be observed that at an SINR of 30 dB, our proposed RA scheme attained a data rate up to 61.5 bps/Hz, whereas RA schemes presented in [5] and [9] attained data rates up to 45 bps/Hz and 41 bps/Hz, respectively. This indicates that the proposed RA scheme maintained a relatively high data rate by efficiently finding the perfectly matched resource reuse partners.

**Figure 5:**
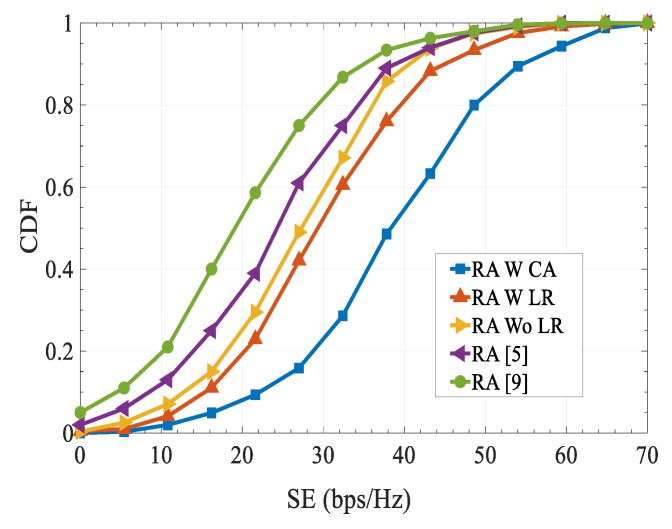
Cumulative Distribution Function (CDF) of spectral efficiency (SE) performance.

The cumulative distribution function of the SE with different schemes is depicted in Figure 5. As can be seen, the proposed RA scheme obtained a higher SE over existing schemes. Specifically, by introducing FFR and combinatorial auction-based matching algorithm together in our proposed RA accommodate the promising preferences of two-sided users and results in an optimal solution. As expected, it can be seen that using our proposed scheme, 80% of users attained a SE of 48.8 bps/Hz. However, RA schemes in [5,9] attain SE of 33.5 bps/Hz and 29 bits/s/Hz, respectively.

**Figure 6:**
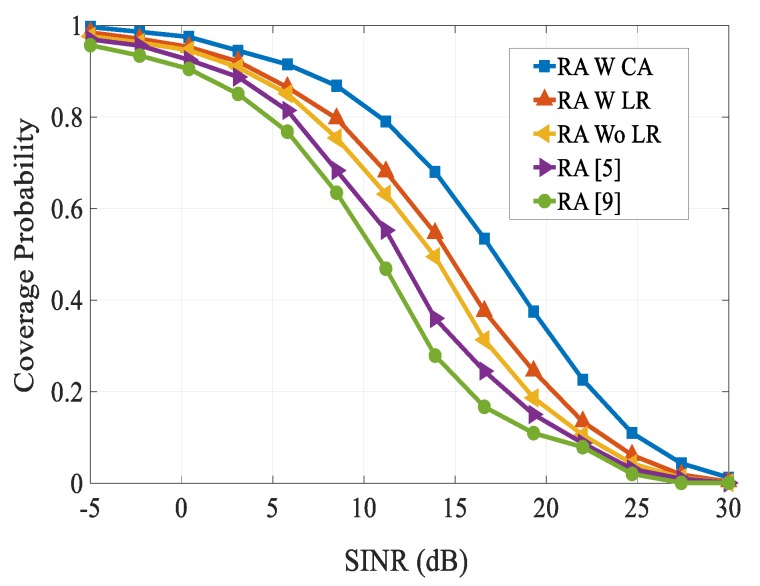
Coverage probability vs. SINR performance.

**Figure 7:**
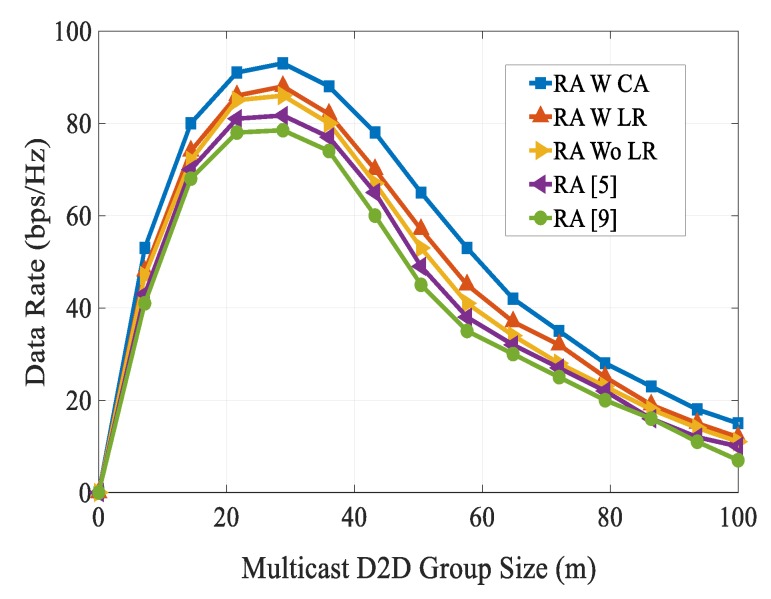
Data rate vs. multicast D2D group size.

Figure 6 shows the comparison of coverage probability of different RA schemes with varying SINR thresholds. As the SINR threshold value increased, the coverage probability decreased due to the path loss effect. We can observe that the coverage probability of our proposed RA scheme was higher than other existing schemes over the entire SINR threshold range considered. From Figure 6, it can be seen that our proposed RA scheme obtained 68% coverage probability at an SINR threshold of 14 dB. However, RA schemes in [5,9] achieved their respective coverage probabilities as 36 dB and 27.8 dB. The data rates had been compared for different multicast D2D group sizes, as shown in Figure 7. We can see that as the multicast D2D cluster size increased up to a certain value, the performance of data rates followed a raising trend and after it reached a peak value of group size, they followed a decreasing trend. Specifically, in the proposed RA scheme, the performance gain increased rapidly when the cluster size was in the range of around 0–30 m, and then started to fall when the cluster size exceeds 30 m. This is because as the multicast D2D group size increased, there was a serious power link fading effect. From Fig., it can be observed that our proposed scheme attained a better performance gain than other schemes over the entire considered multicast cluster sizes.

**Figure 8:**
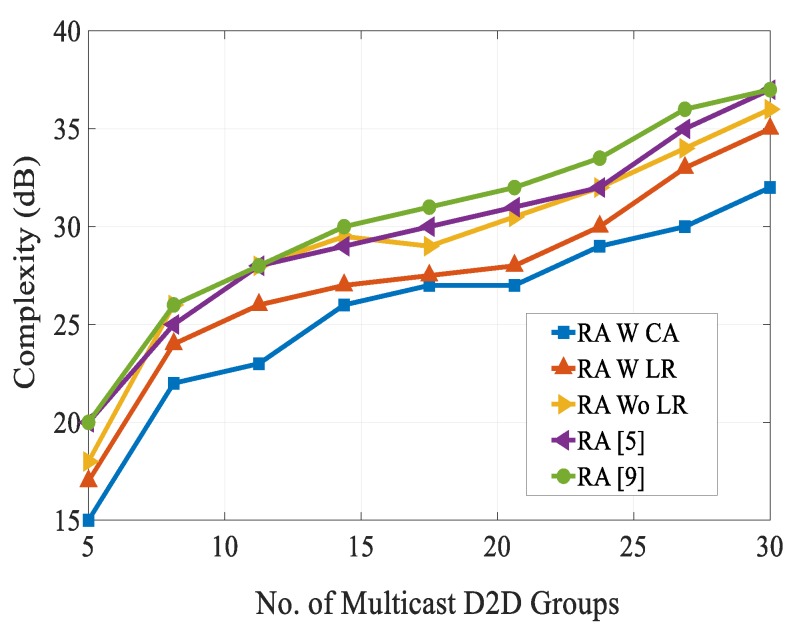
Complexity vs. no. of multicast D2D groups.

**Figure 9:**
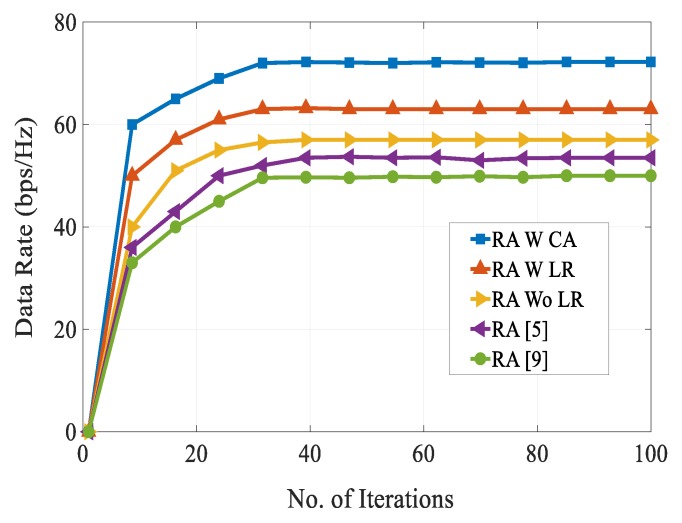
Data rate vs. no. of iterations.

Figure 8 shows the number of multicast D2D groups versus computational complexity for obtaining different SINRs. It can be seen from the figure that the computational complexity of the RA schemes in [5,9] were very high as compared with our proposed RA scheme. This is because the co-channel interference level increased as the number of multicast D2D groups increased in the system. This figure shows the effectiveness of combination of the FFR technique and combinatorial auction-based matching algorithm for multicast D2D cellular networks. At multicast group of 30, our proposed RA scheme attained a complexity of 32 dB as compared with the complexity of 37 dB for the RA schemes presented in [5,9]. Figure 9 shows the data rate of different schemes over the number of iterations. We can see that during different iterations, our proposed RA scheme always resulted in higher data rate than other schemes. This is because of efficient selection of resource reuse partner between the conventional cellular users and multicast D2D users by fulfilling QoS requirements. On the other hand, data rates of the RA schemes presented in [5] and [9] fell due to high co-channel interference and communication overhead.

**Figure 10:**
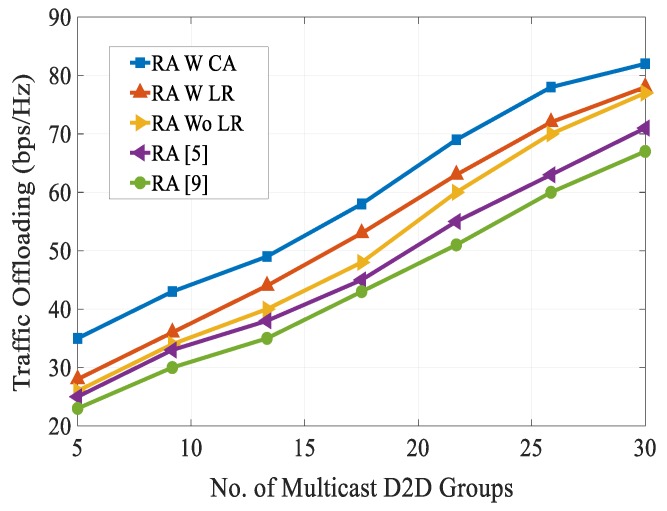
Traffic offloading vs. no. of multicast D2D groups.

**Figure 11:**
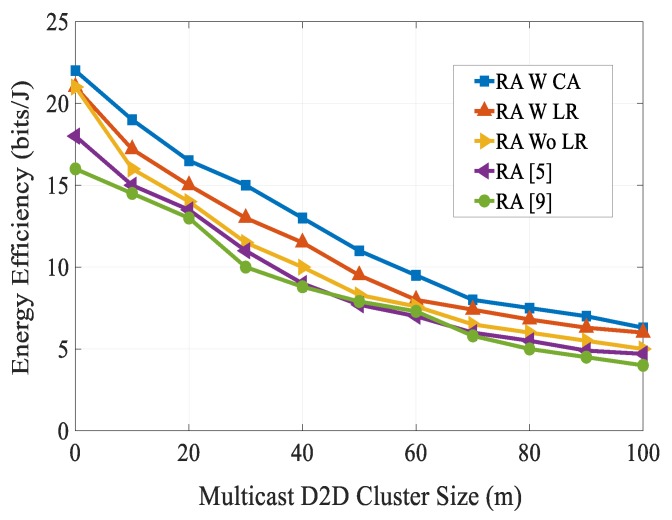
Energy efficiency vs. multicast D2D group size.

Figure 10 shows an analysis of the mobile data traffic offloading with a varying number of multicast D2D groups. It can be seen that the data traffic offloading rose with the increasing number of multicast D2D groups. For the entire considered number of multicast D2D groups, our proposed RA scheme achieved the best traffic offloading rate as compared with other schemes. This is due to the fact that our proposed scheme maintained a tradeoff between QoS requirements and system capacity for both cellular users and multicast D2D users. Figure 11 shows the EE versus the multicast D2D cluster size. From the figure, we can see that the EE performance of all schemes decreased as the multicast D2D cluster size increased. The reason is that as the multicast D2D cluster size increased, there was a need for a higher transmission power to support the required QoS of both cellular users and multicast D2D users. Moreover, as the multicast D2D cluster size increased, the channel gain between the D2D transmitter and corresponding D2D receiver link decreased and co-channel interference became severe. Simulation results show that our proposed RA scheme attained the highest EE over the entire multicast D2D cluster size considered.

**Figure 12:**
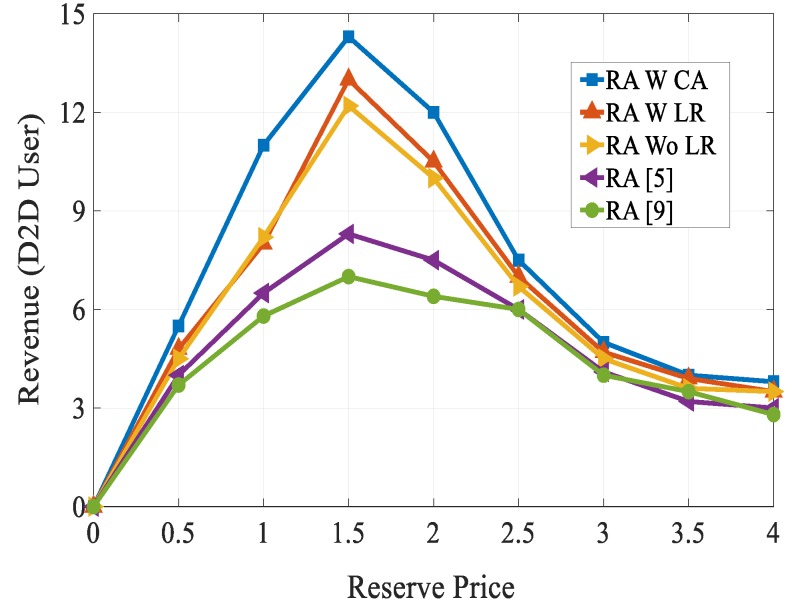
Revenue vs. reserve price.

**Figure 13:**
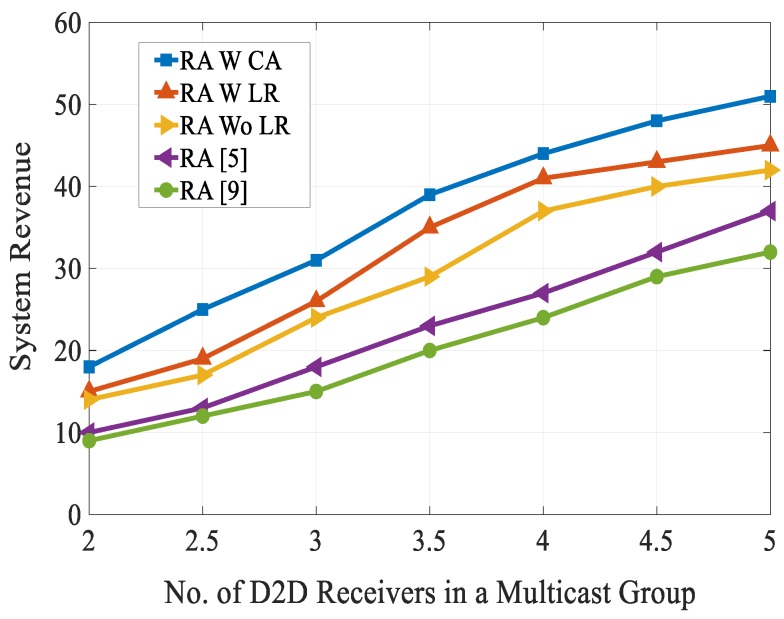
System revenue vs. no. of D2D receivers in a multicast group.

Figure 12 compares the D2D user’s revenue of the proposed algorithm with the state-of-the-art techniques. For analysis, we have set the reserve price between 0 and 4. As can be clearly seen from Figure 12, the D2D user’s revenue increases first till the reserve price is 1.5 and when the reserve price is above 1.5, the revenue of all the schemes decreased. However, the revenue of the proposed algorithm outperformed other schemes. We have calculated the system revenue with varying number of D2D receivers in a multicast group as shown in Figure 13. From Figure 13, we can see that as the number of D2D receivers in the multicast group increased, the system revenue of all the five schemes rose. As expected our proposed combinatorial auction-based matching algorithm outperformed other schemes. This is because a one-to-one bargaining scenario was performed in our proposed scheme, which allowed users to estimate different utility valuations. This phenomenon increased the spectrum resource utilization and system revenue.

**Figure 14:**
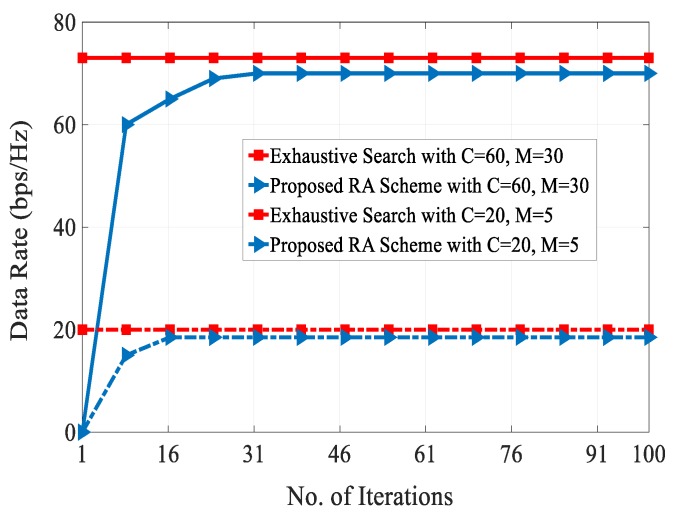
Data rate vs. no. of iterations.

Finally, in order to verify the efficiency of our proposed combinatorial auction-based RA for multicast D2D cellular networks, in Figure 14 we present a data rate performance with varying number of iterations of our proposed scheme and an exhaustive search method. The results show that our proposed RA scheme attains optimal data rate to that of an exhaustive algorithm with much lesser computational complexity.

## 6. Conclusions

The rapid growth of mobile data traffic is causing data congestion problems in existing cellular networks. To handle this problem, multicast D2D uplink cellular networks is proposed. In this work, an FFR scheme with a cell partitioning technique is considered. Then, we formulated throughput optimization problem using the FFR scheme. However, a non-convex scenario is generated in the optimization problem due to the co-channel interference between the conventional cellular users and multicast D2D users. To overcome this issue, we introduced the Lagrange relaxation technique. Furthermore, we proposed a combinatorial auction-based matching algorithm to obtain a scalable and manageable resource allocation. We divide the proposed algorithm into two stages. In the first stage, combinatorial auctioning of available cellular resources is performed based on the bidding prices and in the second stage, we match the resources based on the preferences of the cellular users and multicast D2D users. For analysis, we named cellular users as the seller, multicast D2D users as the buyer, and eNB as the auctioneer. The auctioneer identifies the set of prices from the sellers and collects the bids from the buyers, and then makes a decision that retains the system revenue. Finally, we formulated an optimization problem by guaranteeing the QoS requirements for both cellular users and multicast D2D users. The simulation results showed that the proposed combinatorial auction-based matching algorithm not only offloaded traffic but also obtained a minimum co-channel interference and low computational complexity.

## Figures and Tables

**Table 1:** Main simulation parameters and values.

Parameter	Value
Cellular layout	Seven hexagonal shaped cells
Cell radius (y)	700 m
Multicast D2D group size	(5–100) m
Transmission power of eNB	30 dBm
Maximum Transmission power of CUs	24 dBm
Maximum Transmission power of DUs	20 dBm
Noise spectral density	–174 dBm
Number of cellular users	60
Number of multicast D2D group	5–30
Number of D2D receivers in each multicast group	2–5
Uplink channel Bandwidth	15 MHz
Shadowing standard deviation	8 dB
CU density	0.0001 $m^{-2}$
DU density	0.0004 $m^{-2}$
Path loss exponent	3–5
